# Assessment of the genetic relationship between *Dictyocaulus* species from *Bos taurus* and *Cervus elaphus* using complete mitochondrial genomic datasets

**DOI:** 10.1186/1756-3305-5-241

**Published:** 2012-10-30

**Authors:** Robin B Gasser, Abdul Jabbar, Namitha Mohandas, Johan Höglund, Ross S Hall, D Timothy J Littlewood, Aaron R Jex

**Affiliations:** 1 Department of Veterinary Science, The University of Melbourne, Parkville, Victoria, Australia; 2 Department of Biomedical Sciences and Veterinary Public Health, Section for Parasitology, Swedish University of Agricultural Sciences (SLU), Uppsala, Sweden; 3 Department of Life Sciences, The Natural History Museum, Cromwell Road, London, UK

**Keywords:** *Dictyocaulus* (Nematoda: Strongylida), Lungworms, Dictyocaulosis, Cattle, Deer, Mitochondrial genome, Systematics, Epidemiology

## Abstract

**Background:**

*Dictyocaulus* species are strongylid nematodes of major veterinary significance in ruminants, such as cattle and cervids, and cause serious bronchitis or pneumonia (dictyocaulosis or “husk”). There has been ongoing controversy surrounding the validity of some *Dictyocaulus* species and their host specificity. Here, we sequenced and characterized the mitochondrial (mt) genomes of *Dictyocaulus viviparus* (from *Bos taurus*) with *Dictyocaulus* sp. cf. *eckerti* from red deer (*Cervus elaphus*), used mt datasets to assess the genetic relationship between these and related parasites, and predicted markers for future population genetic or molecular epidemiological studies.

**Methods:**

The mt genomes were amplified from single adult males of *D. viviparus* and *Dictyocaulus* sp. cf. *eckerti* (from red deer) by long-PCR, sequenced using 454-technology and annotated using bioinformatic tools. Amino acid sequences inferred from individual genes of each of the two mt genomes were compared, concatenated and subjected to phylogenetic analysis using Bayesian inference (BI), also employing data for other strongylids for comparative purposes.

**Results:**

The circular mt genomes were 13,310 bp (*D. viviparus*) and 13,296 bp (*Dictyocaulus* sp. cf. *eckerti*) in size, and each contained 12 protein-encoding, 22 transfer RNA and 2 ribosomal RNA genes, consistent with other strongylid nematodes sequenced to date. Sliding window analysis identified genes with high or low levels of nucleotide diversity between the mt genomes. At the predicted mt proteomic level, there was an overall sequence difference of 34.5% between *D. viviparus* and *Dictyocaulus* sp. cf. *eckerti*, and amino acid sequence variation within each species was usually much lower than differences between species. Phylogenetic analysis of the concatenated amino acid sequence data for all 12 mt proteins showed that both *D. viviparus* and *Dictyocaulus* sp. cf. *eckerti* were closely related, and grouped to the exclusion of selected members of the superfamilies Metastrongyloidea, Trichostrongyloidea, Ancylostomatoidea and Strongyloidea.

**Conclusions:**

Consistent with previous findings for nuclear ribosomal DNA sequence data, the present analyses indicate that *Dictyocaulus* sp. cf. *eckerti* (red deer) and *D. viviparus* are separate species. Barcodes in the two mt genomes and proteomes should serve as markers for future studies of the population genetics and/or epidemiology of these and related species of *Dictyocaulus*.

## Background

Species of *Dictyocaulus* (Strongylida: Dictyocaulidae) are economically important parasitic nematodes of the lungs of various ungulate animals, including domestic and wild ruminants (e.g., cattle and deer) [1], and are causative agents of bronchitis and pneumonia (dictyocaulosis or “husk”) [2]. All members of the Dictyocaulidae have direct life cycles [3]. Adult nematodes live in the bronchi, where the ovo-viviparous females produce eggs, from which first stage larvae (L1s) hatch in the lung. The L1s are shed in the faeces from the infected host. Under favourable environmental conditions, the L1s develop through to the infective third-stage larvae (L3s) over a period of ~ 4–6 days. After ingestion by the host, the L3s migrate through the intestinal wall to the mesenteric lymph nodes, moult, and, as fourth stage larvae (L4s), are transported to the lungs. The L4s penetrate the alveoli, moult and then develop into dioecious adults. The period from ingestion to reproductive maturity is estimated at 21–35 days [3].

Since *Dictyocaulus* was first erected [4], there has been ongoing controversy as to the validity of some species within this genus, particularly those infecting bovids and/or cervids [5], because of a lack of reliable morphological features for their unequivocal identification. The identification of *Dictyocaulus* species and populations is not only important from a taxonomic perspective, but also has implications for studying the host and geographical distributions of the parasites, the cross transmissibility of *Dictyocaulus* between or among host species (particularly between bovine and cervid hosts) and also for the control of dictyocaulosis. Although molecular tools, employing genetic markers in ribosomal DNA, have found utility for systematic and/or epidemiological studies of some species [6-13], there is still limited information on the genetic composition of *Dictyocaulus* populations in different ruminant host species and countries around the world*.*

Mitochondrial (mt) genomes provide markers for systematic, genetic and epidemiological investigations of species of strongylid nematodes, with a perspective on discovering population variants and cryptic species and exploring transmission patterns linked to particular genotypes of a species [14-16]. For instance, we have shown that not only are mt genomic regions useful for exploring the population genetic structures of *Dictyocaulus viviparus*[12,17], concatenated amino acid sequences inferred from whole mt genomes can provide barcodes for nematodes [16]. Although nucleotide sequence variation can be considerable (≤3.5%; upon pairwise comparison) in some of the protein coding mt genes of *D. viviparus* studied to date [12], the inferred amino acid sequence variation is less. For example, previous studies have shown amino acid sequence variation of 0–1.5% (over 131 positions in COX1) (among 252 individual worms) [17], of 0–1.6% for COX3 (over 125 positions) and 0–2.3% for NAD5 (over 132 positions) (72 individual worms) [12]. Importantly, current evidence indicates that concatenated amino acid sequence datasets can be employed for the retesting of hypotheses regarding the systematic relationships of nematodes; such sequence datasets are relatively large and usually have excellent phylogenetic signal, often achieving nodal support values of 98-100% in tree reconstructions [16,18]. High throughput sequencing and new computational approaches [19] have underpinned these advances, and now enable mt proteomic barcodes to be defined for *Dictyocaulus* species from a range of ungulate hosts. Here, as a first step, we used a massively parallel sequencing method and semi-automated bioinformatic pipeline for the characterisation of the mt genomes of *D. viviparus* (from *Bos taurus*) and *Dictyocaulus* sp. cf. *eckerti* (from *Cervus elaphus*) (cf. [20]), which we compared directly with those of other lungworms, for which published whole mt genomic datasets were available [16,21]. We also studied the genetic relationships between these two *Dictyocaulus* species and selected representatives of the order Strongylida, and identified regions in the mt genomes of *Dictyocaulus* that might serve as markers for future population genetic or molecular epidemiological studies.

## Methods

### Parasites, DNA isolation and identification

Adult specimens of *Dictyocaulus* were collected from the bronchi of the lungs of cattle (from Sweden) or red deer (from New Zealand) in previous studies [17,20,22,23]. The worms originally collected were washed extensively in physiological saline and then stored at −80 °C until use. Upon thawing, the anterior and posterior ends of each nematode were cut off and cleared in lactophenol for subsequent morphological identification. The mid-body section of each worm was used for the extraction of genomic DNA using a small-scale sodium dodecyl-sulphate (SDS)/proteinase K digestion and mini-column purification (Wizard DNA Clean-Up Kit, Promega, USA) [23]. The molecular identity of each specimen was verified by PCR-based sequencing of the second internal transcribed spacer (ITS-2) of nuclear ribosomal DNA (rDNA) using an established method [23].

### Sequencing and assembly of mt genomes

Using the protocol described by Hu et al. [24], the complete mt genome was amplified by long-PCR (BD Advantage 2, BD Biosciences) as two overlapping amplicons (~5 kb and ~10 kb) from the genomic DNA from the mid-body section of a single male specimen of *D. viviparus* from *B. taurus* and of *Dictyocaulus* sp. cf. *eckerti* from *C. elaphus*. Amplicons were consistently produced from the positive control samples (total genomic DNA of *Angiostrongylus vasorum*); in no case was a product detected for any of the negative (no-template) controls. Amplicons were then treated with shrimp alkaline phosphatase and exonuclease I, and quantified in a spectrophotometer (ND-1000 UV–VIS v.3.2.1, NanoDrop Technologies). Following an electrophoretic analysis of quality, the two amplicons (2.5 μg of each) from each of the two worms from each host species were pooled and then sequenced using the 454 Genome Sequencer FLX (Roche) [25]. The mt genome sequences (GenBank accession nos. JX519459 and JX519460) were each assembled from (~300 bp) reads using the program CAP3 [26].

### Annotation and analysis of mt genomic sequence data

The genes and features of each mt genome were annotated using an established computational pipeline [16]. In brief, each protein-encoding mt gene was identified by local alignment comparison (six reading frames) using amino acid sequences conceptually translated from corresponding genes from the mt genome of a reference species (e.g., *Metastrongylus pudendotectus*; accession no. GQ888714; [16]). The large and small subunits of the mt *rrn* (ribosomal RNA = rRNA) genes (*rrn*S and *rrn*L, respectively) were identified by local alignment of nucleotide sequence data. The *trn* (transfer RNA = tRNA) genes were predicted (from both strands) according to their structure, using scalable models, based on the standard nematode mt tRNAs [15]. All predicted *trn* genes were then grouped, based on their anti-codon sequence, and identified based on the amino acid encoded by this anti-codon. Two separate *trn* gene groups were predicted each for serine (one each for the anticodons AGN and UCN, respectively) and leucine (one each for the anticodons CUN and UUR, respectively), because these *trn* genes are duplicated in many invertebrate mt genomes [15]. All predicted tRNAs within each amino acid group were ranked based on structural “strength” (as inferred by the number of nt mismatches in each stem), and the 100 best-scoring structures for each group were compared by BLASTn against a custom database representing all published nematode mt genome sequences available in the GenBank database (accessible *via*http://www.ncbi.nlm.nih.gov).

All *trn* genes of each mt genome were then identified and annotated based on maximum sequence identity to known nematode tRNAs. Annotated sequence data were imported using the program SEQUIN (available *via*http://www.ncbi.nlm.nih.gov/Sequin/) for the final verification of the mt genome organization/annotation prior to submission to the GenBank database.

Sliding window analysis was performed on the aligned, complete mt genome sequences of the two *Dictyocaulus* species using DnaSP v.5 [27]. The alignment of these sequences was achieved using the program MUSCLE v.3.8 [28], as implemented in SeaView v.4 [29]. Keeping the nucleotides in frame, there were no ambiguously aligned regions. A sliding window of 300 bp (steps of 10 bp) was used to estimate nucleotide diversity (π) over the entire alignment; indels were excluded using DnaSP. Nucleotide diversity for the entire alignments was plotted against midpoint positions of each window, and gene boundaries were defined.

### Alignment and phylogenetic analysis of concatenated amino acid sequence data

The amino acid sequences conceptually translated from individual genes of each of the two mt genomes (representing *D. viviparus* and *Dictyocaulus* sp. cf. *eckerti*) were concatenated. Selected for comparison were concatenated amino acid sequences predicted from published mt genomes from key nematodes representing the order Strongylida, including *M. pudendotectus, M. salmi, Angiostrongylus cantonensis* and *A. costaricensis* (accession nos. GQ888714, GQ888715, GQ398121 and GQ398122, respectively; superfamily Metastrongyloidea), *Haemonchus contortus* (NC_010383; Trichostrongyloidea), *Ancylostoma caninum* (FJ483518; Anyclostomatoidea) and *Oesophagostomum dentatum* (GQ888716; Strongyloidea) [16,21]. All amino acid sequences (considering all homologous characters) were aligned using MUSCLE [28] and then subjected to phylogenetic analysis using Bayesian inference (BI), essentially as described by Jex et al. [16], employing an unconstrained model prior (prset aamodelpr = mixed). The tree was functionally rooted against *Metastrongylus* species, the earliest divergent metastrongyloid in Jex et al. [16].

## Results

### Features of the mt genomes of *D. viviparus* and *Dictyocaulus* sp. cf. *eckerti* from red deer

**Figure 1:**
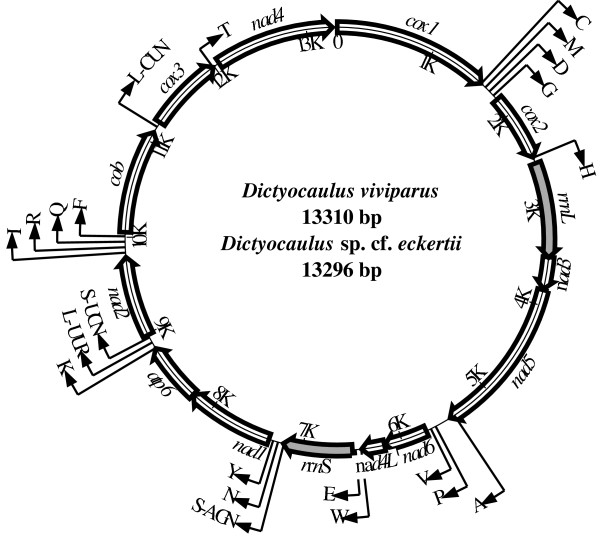
**Schematic representation of the circular mt genome of *Dictyocaulus viviparus *and *Dictyocaulus *sp. cf. *eckerti*. **Each transfer RNA gene is identified by a one-letter amino acid code and ribosomal RNA genes are shaded.

The circular mt genome sequences determined for *D. viviparus* and *Dictyocaulus* sp. cf. *eckerti* were 13,310 bp and 13,296 bp in size (Figure 1), being similar to those published for *Metastrongylus pudendotectus* (13,793 bp) and *M. salmi* (13,778 bp) [16]. The nucleotide compositions of the two *Dictyocaulus* mt genomes were similar, being ~ 24.6% for A, 6.3% for C, 17.3% for G and 51.8% for T (Table 1). As expected, these two mt genomes were AT-rich, with T and C being the most and least favoured nucleotides, respectively. Both mt genomes contained genes encoding 12 proteins (COX1-3, NAD1-6, NAD4L, ATP6 and CYTB), two *rrn* and 22 *trn* genes but, as expected (cf. [15]), lacked an *atp*8 gene. All genes were inferred to be transcribed in the same direction (5’ > 3’) (Figure 1) and the gene arrangement (GA2) was the same as that of other strongylid nematodes [15].

**Table 1:** **Nucleotide composition (%) of the entire mt genome of *Dictyocaulus viviparus* (*Dv*) and *Dictyocaulus *sp. cf. *eckerti *(*De*), and of protein and RNA genes within this genome**

**Lungworm**	**Nucleotide**	**Length (bp)**	**A**	**C**	**G**	**T**	**A + T**
*Dv*	Entire sequence	13310	24.6	6.3	17.3	51.8	76.4
	Protein genes	10143	21.6	6.7	18.6	53.1	74.7
	RNA genes	1659	33.5	5.3	14.0	47.2	80.7
*De*	Entire sequence	13296	24.7	6.3	17.4	51.6	76.3
	Protein genes	9807	21.9	6.9	18.6	52.6	74.5
	RNA genes	1659	33.9	5.6	14.3	46.2	80.1

### Protein genes and codon usages

The initiation and termination codons predicted for the protein encoding genes of *D. viviparus* were compared with those of *Dicyocaulus* sp. cf. *eckerti* (Table 2). The commonest start codon for *D. viviparus* was ATT and TTG (for three of 12 proteins each), followed by ATA and GTT (two genes), ATC and TTA (one gene each); for *Dictyocaulus* sp. cf. *eckerti*, it was ATA (four genes), followed by TTG (three genes), ATG (two genes), ATT, GTT and TTA (one gene each). Eight mt protein genes in *D. viviparus* and nine in *Dictyocaulus* sp. cf. *eckerti* had a TAA or TAG translation termination codon; the other protein genes ended in an abbreviated stop codon, such as TA or T (Table 2). For both *Dicyocaulus* species, the 3'-ends of these genes were immediately adjacent to a downstream *trn* gene (Figure 1; Table 2), consistent with the arrangement for *M. salmi*[16].

**Table 2:** **Locations and lengths of protein encoding genes in the mt genome of *Dictyocaulus viviparus (Dv) *and *Dictyocaulus eckerti (De) *as well as their initiation and termination codons and the lengths of the predicted proteins**

**Gene**	**Positions and nucleotide sequence lengths (bp)**		**Initiation and Termination codons and the amino acid sequence lengths**	
	***D. viviparus***	***D. eckerti***	***D. viviparus***	***D. eckerti***
*cox*1	1-1579 (1578)	1-1573 (1572)	ATC-TAA (525)	TTG-TAA (523)
*trn*C	1580-1634 (54)	1574-1629 (55)		
*trn*M	1636-1693 (57)	1630-1686 (56)		
*trn*D	1693-1749 (56)	1686-1743 (57)		
*trn*G	1749-1806 (57)	1744-1798 (54)		
*cox*2	1806-2490 (684)	1795-2482 (687)	ATT-TAG (227)	TTA-TAG (228)
*trn*H	2491-2549 (58)	2483-2541 (58)		
*rrn*L	2555-3516 (961)	2549-3508 (959		
*nad*3	3502-3835 (333)	3482-3800 (318)	GTT-TAA (110)	TTG-TAG (105)
*nad*5	3844-5414 (1570)	3822-5388 (1566)	GTT-T (523)	GTT-T (522)
*trn*A	5414-5469 (55)	5389-5444 (55)		
*trn*P	5631-5686 (55)	5603-5659 (56)		
*trn*V	5684-5739 (55)	5657-5712 (55)		
*nad*6	5739-6174 (435)	5728-6142 (414)	TTG-TAG (144)	ATA-TAA (137)
*nad*4L	6181-6415 (234)	6129-6383 (255)	ATT-T (77)	ATT-T (84)
*trn*W	6413-6469 (56)	6376-6432 (56)		
*trn*E	6469-6527 (58)	6432-6490 (58)		
*rrn*S	6504-7200 (696)	6471-7165 (694)		
*trn*S (UCN)	7191-7256 (65)	7166-7219 (53)		
*trn*N	7253-7306 (53)	7218-7272 (54)		
*trn*Y	7304-7359 (55)	7269-7325 (56)		
*nad*1	7350-8232 (882)	7316-8138 (822)	TTG-T (271)	ATA-TAG (273)
*atp*6	8234-8831 (597)	8231-8813 (582)	ATT-TAA (199)	ATA-TAA (193)
*trn*K	8832-8891 (59)	8815-8874 (59)		
*trn*L (UUR)	8898-8954 (56)	8875-8930 (55)		
*trn*S (AGN)	8955-9005 (50)	8931-8988 (57)		
*nad*2	8986-9802 (816)	8989-9812 (823)	TTG-T (271)	ATA-TAG (273)
*trn*I	9842-9897 (55)	9824-9880 (56)		
*trn*R	9897-9965 (68)	9881-9935 (54)		
*trn*Q	9952-10006 (54)	9936-9990 (54)		
*trn*F	10006-10064 (58)	9990-10047 (57)		
*cob*	10050-11103 (1053)	10034-11084 (1050)	ATA-T (350)	ATG-T (349)
*trn*L (CUN)	11161-11215 (54)	11142-11197 (55)		
*cox*3	11216-11987 (771)	11197-11989 (792)	TTG-TAA (256)	TTG-TAA (263)
*trn*T	11982-12034 (52)	11963-12019 (56)		
*nad*4	12038-13262 (1224)	12077-13244 (1167)	TTA-TAG (407)	ATA-TAG (388)

Gene abbreviations are according to [69].

The codon usage for the 12 protein genes of *D. viviparus* was also compared with *Dictyocaulus* sp. cf. *eckerti* (Table 3); 62 of the 64 possible codons were used. Neither codon CGC (Arg) nor CTC (Leu) were utilized in the mt genome of *D. viviparus*. Codons CGC (Arg) and GAC (Asp) were not utilized in the mt genome of *Dictyocaulus* sp. cf. *eckerti*. The preferred nucleotide usage at the third codon position of mt protein genes of *D. viviparus* and *Dictyocaulus* sp. cf. *eckerti* reflects the overall nucleotide composition of these mt genomes. At this position, T is the most frequently, and C the least frequently used. For *D. viviparus* and *Dictyocaulus* sp. cf. *eckerti*, the codons ending in A have higher frequencies than the codons ending in G, which is similar to, for example, other members of the order Strongylida and *Caenorhabditis elegans* (Rhabditida), but distinct from *Ascaris suum* (Ascaridida) and *Onchocerca volvulus* (Spirurida) (accession nos. in [15]). As the usage of synonymous codons is proposed to be preferred in gene regions of functional significance, codon bias might be linked to selection at silent sites [30,31].

**Table 3:** **Number of codons and codon usages (%) in mt protein genes of *Dictyocaulus viviparus *(*Dv*) and *Dictyocaulus *sp. cf. *eckerti *(*De*)**

**Amino acid**	**Codon**	***Dv***	***De***
Non-polar			
Alanine	GCN	59 (1.7)	60 (1.8)
Isoleucine	ATY	185 (5.5)	192 (5.9)
Leucine	CTN	37 (1.1)	43 (1.3)
Leucine	TTR	492 (14.6)	476 (14.6)
Methionine	ATR	175 (5.2)	191 (5.8)
Phenylalanine	TTY	589 (17.4)	550 (16.8)
Proline	CCN	71 (2.1)	63 (1.9)
Tryptophan	TGR	71 (2.1)	75 (2.3)
Valine	GTN	292 (8.6)	288 (8.8)
Polar			
Aspargine	AAY	121 (3.6)	121 (3.7)
Cysteine	TGY	57 (1.7)	72 (2.2)
Glutamine	CAR	32 (0.9)	31 (0.9)
Glycine	GGN	191 (5.7)	177 (5.4)
Serine	AGN	165 (4.9)	158 (4.8)
Serine	TCN	165 (4.9)	173 (5.3)
Threonine	ACN	90 (2.7)	81 (2.5)
Tyrosine	TAY	196 (5.8)	191 (5.8)
Acidic			
Aspartate	GAY	69 (2.0)	70 (2.1)
Glutamate	GAR	73 (2.2)	63 (1.9)
Basic			
Arginine	CGN	35 (1.0)	30 (0.9)
Histidine	CAY	49 (1.4)	50 (1.5)
Lysine	AAR	107 (3.2)	101 (3.1)

International Union of Pure and Applied Chemistry (IUPAC) codes (N = A, G, C or T; Y = C or T; R = A or G) were used.

AT bias in the nucleotide composition was also reflected in a bias in the amino acid composition of proteins. The AT-rich codons represent the amino acids Phe, Ile, Met, Tyr, Asn or Lys, and the GC-rich codons represent Pro, Ala, Arg or Gly. In the mt genomes of *D. viviparus* and *Dictyocaulus* sp. cf. *eckerti*, the most frequently used codons are TTT (Phe), TTA (Leu), ATT (Ile), TTG (Leu), TAT (Tyr) and GTT (Val). The least frequently used codons were CTA, CTG (Leu), ATC (Ile), GTC (Val), AGC (Ser), CCC (Pro), GCC (Ala), TAC (Tyr), CAC (His), AAC (Asn), CGA (Arg), TCC (Ser) and GGC (Gly). All four GC-rich only codons are represented here, and every codon had at least one C. When the frequencies of synonymous codons within the AT-rich group, such as Phe (TTT, 16.1% and 16.2%; TTC, 0.7% and 0.3%), Ile (ATT, 5.7% and 5.3%; ATC, 0.15% and 0.03%), were compared between both mt genomes, the frequency was always less if the third position was a C.

### Transfer RNA genes

Twenty-two *trn* genes were identified in the mt genomes of both *D. viviparus* and *Dictyocaulus* sp. cf. *eckerti*. The *trn* gene sequences ranged from 50–68 nt in length. The *trn* structures had a 7 bp amino-acyl arm, a 3–4 bp DHU arm, a 4–5 bp anticodon stem, a 7 base anticodon loop, with a T always preceding an anticodon and a purine always following an anticodon. Twenty of the 22 *trn* genes (i.e. excluding the two *trn*S genes) had a predicted secondary structure with a 3–4 bp DHU stem and a DHU loop of 5–8 bases, in which the variable TψC arm and loop were replaced by a “TV-replacement loop” of 6–12 bases, in accordance with other nematodes [15]. The mt *trn*S of both *Dictyocaulus* mt genomes had a secondary structure consisting of a DHU replacement loop of 4–6 bases, 3 bp TψC arm, TψC loop of 6–9 bases and a variable loop of 4 bases, consistent with other nematodes of the class Secernentea [32,33], but distinct from *Trichinella spiralis* and *Trichuris suis* (class Adenophorea) [34,35].

### Ribosomal RNA genes

The *rrn*S and *rrn*L genes of each of the two *Dictyocaulus* species were identified by sequence comparisons with homologous sequences of *A. cantonensis*. The *rrn*S gene was located between *trn*E and *trn*S (UCN), and *rrn*L was between *trn*H and *nad*3. The two genes were separated from one another by the protein genes *nad*3, *nad*5, *nad*6 and *nad*4L (Figure 1). The sizes of the *rrn*S genes of *D. viviparus* and *Dictyocaulus* sp. cf. *eckerti* were 696 and 694 bp, respectively. The sizes of the *rrn*L genes of *D. viviparus* and *Dictyocaulus* sp. cf. *eckerti* were 961 bp and 959 bp, respectively. The lengths of these two genes were similar to those of other lungworms for which mt genomes have been characterised (696–699 bp for *rrn*S, and 958–961 bp for *rrn*L; [16,21]). The AT contents of the *rrn* genes for *D. viviparus* and *Dictyocaulus* sp. cf. *eckerti* were 80.7% and 80.1%, respectively (Table 1). The overall percentage of identity in *rrn*S sequence between the two species was 77.3%, whereas for of *rrn*L, the identity was 75.8%.

### Sequence comparisons and genetic relationships of *D. viviparus* and *Dictyocaulus* sp. cf. *eckerti* with selected strongylids

**Figure 2:**
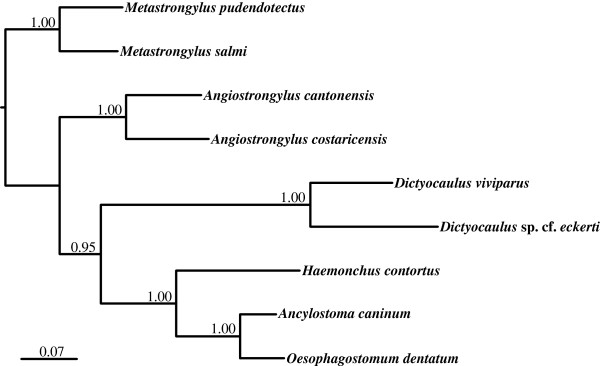
**Relationship of *Dictyocaulus viviparus *with *Dictyocaulus *sp. cf. *eckerti *and selected species representing different superfamilies of the order Strongylida, including *Metastrongylus pudendotectus, M. salmi (Metastrongyloidea), Haemonchus contortus *(Trichostrongyloidea)**, ***Oesophagostomum dentatum *(Strongyloidea) and *Ancylostoma caninum *(Ancylostomatoidea), based on a phylogenetic analysis of concatenated amino acid sequence data for the 12 mt proteins by Bayesian inference.** Posterior probabilities are indicated at each node. Scale bar represents number of substitutions per site.

The amino acid sequences predicted from individual protein-encoding mt genes of *D. viviparus* were compared with those of *Dictyocaulus* sp. cf. *eckerti* (Table 4). Pairwise comparisons of the concatenated sequences revealed identities of 63.0-96.6% (65.5% overall) between them. Based on identity, COX-1 was the most conserved protein, whereas NAD6 and ATP6 were the least conserved (see Table 4). Phylogenetic analysis of the concatenated amino acid sequence data for the 12 mt proteins showed that both *D. viviparus* and *Dictyocaulus* sp. cf. *eckerti* were closely related, and grouped (pp = 1.00) to the exclusion of *Metastrongylus* and *Angiostrongylus* species (Metastrongyloidea) as well as *H. contortus* (Trichostrongyloidea), *An. caninum* (Ancylostomatoidea) and *O. dentatum* (Strongyloidea) (Figure 2).

**Table 4:** **Amino acid sequence identities in the inferred mt proteins between *Dictyocaulus viviparus *(*Dv*) and *Dictyocaulus *sp. cf. *eckerti *(*De*)**

**Protein**	**Identity between *Dv* and *De* (%)**
ATP6	64.0
COB	84.8
COX1	96.6
COX2	83.3
COX3	90.0
NAD1	73.7
NAD2	68.5
NAD3	66.3
NAD4	80.1
NAD4L	71.8
NAD5	77.6
NAD6	63.0
All proteins (concatenated)	65.5

Gene product abbreviations are according to [69].

### Nucleotide variation between the mt genomes of *D. viviparus* and *Dictyocaulus* sp. cf. *eckerti*

**Figure 3:**
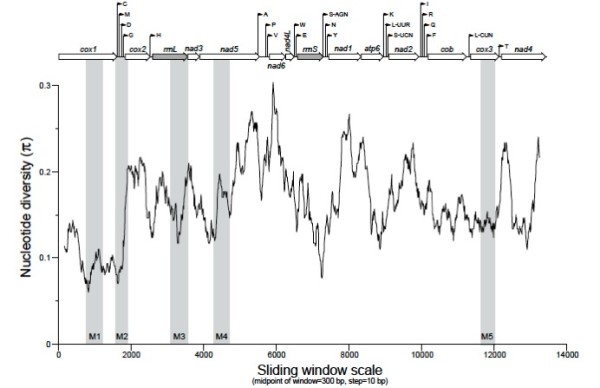
**Sliding window analysis of complete mt genome sequences of *Dictyocaulus viviparus *and *Dictyocaulus *sp. cf. *eckerti*. **The black line indicates nucleotide diversity in a window size of 300 bp, with a step size of 10 bp. Individual pairwise results are shown by symbols (see key). Gene boundaries indicated with shaded columns of gene regions used in previous studies [12,17].

Nucleotide diversities calculated from pairwise comparisons across the mt genomes of *D. viviparus* and *Dictyocaulus* sp. cf. *eckerti,* achieved by sliding window analyses, are shown in Figure 3. Also indicated on the graph are the regions of *cox*1 (452 bp), and of *trn*C_M_D_G (320 bp), *rrn*L (484 bp), *nad*5 (426 bp) and *cox*3 (401 bp) used previously as markers to assess genetic diversity within *D. viviparus* populations [12,17]. In Figure 3, these markers are indicated with M1, M2, M3, M4 and M5, respectively, and lengths include PCR primers. Greatest nucleotide diversity was detected within *nad*6, followed by peaks of variation within *nad*5, *nad*1 and *atp*6. Gene-by-gene nucleotide diversity was highly variable, but, by far, the least variation was recorded within *cox*1. Of the markers used previously by Hu et al. [17] (i.e. M1) and Höglund et al. [12] (i.e. M2-M5), M1 and M2 (within *cox*1) captured the least, and M4 (within *nad*5) captured the greatest sequence variation. The relatively low variation captured by targeting *trn* genes is also indicated by a trough for M2. Overall, the full sliding window indicates a wealth of new genes capable of providing high levels of nucleotide variation for population genetic studies.

## Discussion

Advanced molecular methods are having widespread impact and implications in parasitology [36-47]. In the present study, we utilised massively parallel sequencing and semi-automated bioinformatic annotation for the characterisation of the complete mt genomes of *D. viviparus* (from *Bos taurus*) and *Dictyocaulus* sp. cf. *eckerti* from red deer, and explored the genetic relationships of these two lungworms and selected representatives of the Strongylida. Using sliding window analysis (Figure 3), we also identified regions in these mt genomes which might serve as suitable markers for future molecular explorations of the systematics, population genetics or epidemiology of *Dictyocaulus* species.

Given the controversy surrounding the taxonomy/systematics of some species of *Dictyocaulus*[1,5,8,9,48-54], concatenated mt proteomic sequences might be applied effectively as barcodes to genetically characterise and compare dictyocaulids from various ungulate hosts, including domestic and wild bovids and cervids. This is particularly pertinent, given that *Dictyocaulus* from red deer is genetically distinct from *Dictyocaulus* species from roe deer (*Capreolus capreolus*), fallow deer (*Dama dama*) and moose (*Alces alces*) [7,8,54]. Current evidence shows a distinct genetic differentiation between *Dictyocaulus* sp. cf. *eckerti* and *D. viviparus*, supported by previous results for the ITS-2 region [10]. A detailed appraisal of previously published findings for *D. viviparus*[12,17] revealed a maximum nucleotide sequence variation of 3%, 3.5% and 3%, respectively, in partial *cox*1, *cox*3 or *nad*5 regions (375–396 nt) among 72–252 individual worms. These levels of within-species variation are much lower than estimated levels of difference (9%, 15% and 18%, respectively) between *Dictyocaulus* sp. cf. *eckerti* and *D. viviparus* for the same gene regions (this study). At the amino acid level, variation in COX-1 (131 amino acids) within *D. viviparus* was ≤1.5%, but there was no amino acid difference between *Dictyocaulus* sp. cf. *eckerti* and *D. viviparus* (because of relative conservation). This contrasts the situation for COX3 (125 amino acids) and NAD5 (132 amino acids), for which maximum variation within *D. viviparus* was 1.6% and 2.3%, respectively (cf. [12]), and differences between *D. viviparus* and *Dictyocaulus* sp. cf. *eckerti* were substantially higher, at 10.4% and 13.6%, respectively. Together with previous ITS-2 data [10], this information indicates that *Dictyocaulus* sp. cf. *eckerti* and *D. viviparus* are separate species.

Whether the parasite from red deer (i.e., *Dictyocaulus* cf. *eckerti*) represents a distinct species from (or a population variant of) “*D. eckerti*” (cf. [50]) remains to be established. A previous study showed that the magnitude of sequence difference (~6-8%) in ITS-2 rDNA of *Dictyocaulus* from fallow deer from Germany [7] is greater than variation (0.4-2.6%) among selected individuals of *Dictyocaulus* from red deer [10], although the degree of genetic variation within the operational taxonomic unit from fallow deer is not yet known. *D. eckerti* was originally described from the reindeer, *Rangifer tarandus*, from Western Siberia (cited in [50]), which raises questions as to the specific identity of *Dictyocaulus* from various cervid species [5,8,13,55]. Although Durette-Desset et al. [5] proposed that dictyocaulids of European cervids be called *Dictyocalus noerneri*, molecular and morphological data have shown that roe deer and moose can harbour *D. capreolus* (e.g., [8,54]) and that chamois (*Rupicapra rupicapra*) might harbour a unique species [13]. This controversy and the findings of numerous previous studies emphasize the need for detailed investigations of *Dictyocaulus* specimens from various species of wild and domestic bovid and cervid hosts from different continents, which could be achieved using a combined morphological and mt proteomic barcoding approach.

There is also significance in using mt genetic markers for studying the genetic composition of populations of *Dictyocaulus* spp., given that there are few morphological features for the specific differentiation of some developmental stages (i.e., larvae) [3] and given that cryptic species have been detected within the Strongylida [56,57]. In nematodes, mt DNA is usually more variable in sequence within a species than ITS-2 and other rDNA regions [56], indicating that mt gene regions are well suited for studying the population genetics of parasitic nematodes [14,56,58,59]. The sliding window analysis conducted herein displayed distinct patterns of nucleotide diversity between the two mt genomes representing *Dictyocaulus* (Figure 3). Low variability is useful for the design of oligonucleotide primers that flank mt regions with high variability for population genetic or epidemiological investigations. Some previous studies have shown the utility of some mt gene regions. For instance, Hu et al. [17] employed primer sets, originally designed to *cox*1 of flatworms [60], for PCR-based mutation scanning and selective sequencing analysis of *D. viviparus* individuals from 17 different populations (farms). Within-species variation in *cox*1 was low (0.3–2.3%) [17], similar to findings for some parasitic nematodes of plants and insects [61-65] but distinctly different from findings for gastrointestinal trichostrongyloid nematodes of domestic ruminants [66,67]. In the present study, *cox*1 is revealed to be the gene with the lowest nucleotide diversity between the two *Dictyocaulus* species. In another study, Höglund et al. [12] showed distinct genetic substructuring in *D. viviparus* populations from Sweden using four mt regions (in the *cox3, nad5, trn*C_M_D_G and *rrn*L genes) covering 11% (1542 bp) of the mt genome of *D. viviparus* (Figure 1). Although some of the gene regions (e.g., *cox*3 and *nad*5) used to date provided some resolution of genetic variation (<6% among haplotypes, upon pairwise comparison), the present sliding window analysis indicates other candidate genes for capturing greater diversity. That the *cox*1 region (of 393 bp) is the most conserved relative to other regions (see Figure 3; M1) is a feature that facilitates the design of primers for PCR, but might come at the cost of missing signal (of sequence variation) required for analysis. Here, numerous sequence tracts in the mt genome have been identified as potentially suitable for population genetic or taxonomic studies (Figure 3). The comparison among the mt genomes of different species of nematodes characterised to date now provides the prospect for designing generic or specific primers to capture regions of variability most suited to the resolution needed for analysis, and also provides opportunities for a range of DNA amplification-based approaches, including multiplexed diagnostic methods (cf. [68]).

PCR primers can be designed rationally in conserved regions flanking “variable sequence tracts” within the mt genome considered to be most informative for population genetic studies of *Dictyocaulus* from different bovid and cervid hosts. Utilizing such primer sets, PCR-based single-strand conformation polymorphism (SSCP) analysis [23] could be applied to screen large numbers of individual specimens representing different host species and populations for haplotypic variability (at any developmental stage). A previous study has shown the merit of SSCP for exploring the genetic variation in various populations of *D. viviparus* in Sweden [17], and could be applied to large-scale studies of *Dictyocaulus* specimens representing distinct operational taxonomic units (based on ITS-2) and different ungulate host species.

Using a range of variable mt regions, in combination with classical parasitological techniques, it might also be possible to assess the cross transmission of particular species or genetic variants (haplotypes) of *Dictyocaulus* between/among cattle and cervid hosts, and their pathogenicity in different host species (cf. [22]). Furthermore, it will also be significant to extend mt genome sequencing to a range of lungworm species of domestic and wild ruminants, and assess their genetic relationships. The present study shows the relevance of the mt genomes of *Dictyocaulus* species for future systematic investigations and, importantly, stimulates a reassessment of the phylogenetic position of the family Dictyocaulidae in relation to the Metastrongylidae, Trichostrongylidae and other families within the order Strongylida (cf. [11]).

## Conclusions

Comparative analyses of proteomic sequence datasets inferred from the mt genomes of *Dictyocaulus* sp. cf. *eckerti* (red deer) and *D. viviparus* indicate that these parasites are closely related species. Barcodes identified in the mt genomes and proteomes could serve as markers for future studies of the systematics, population genetics and/or epidemiology of a range of *Dictyocaulus* species as well as other lungworms.

## Competing interests

The authors declare that they have no competing interests.

## Authors’ contributions

RBG, ARJ & DTJL conceived the project and attracted the funding; AJ, ARJ, NM & JH carried out molecular laboratory work; NM, ARJ, DTJL, RSH & RBG carried out data analysis and interpretation; RBG, ARJ & DTJL wrote the draft manuscript with critical input from AJ and JH. All authors read and approved the final version of the manuscript.
